# Supplementary figures and images for: Combinative effects of β-elemene and propranolol on the proliferation, migration, and angiogenesis of hemangioma
Source: PeerJ. 2023 Jul 12;11:e15643. doi: 10.7717/peerj.15643 (PMC10349565; doi:10.7717/peerj.15643)

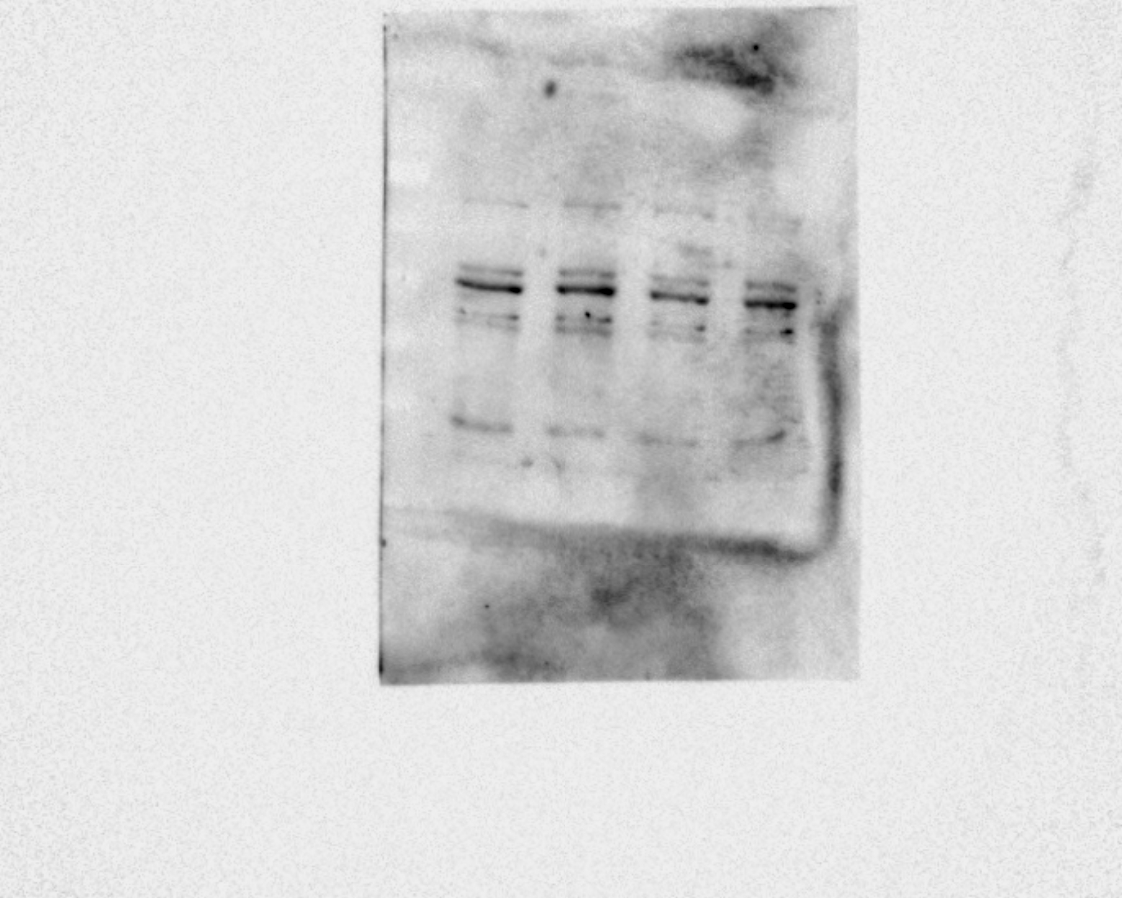

Supplement: Data S1 [file peerj-11-15643-s001.zip › ╨┬╜¿╬─╝■╝╨/(Ctrl,a┬-elemene,propranolol,a┬-elemene+propranolol) Akt,Erk,GAPDH(second).jpg]

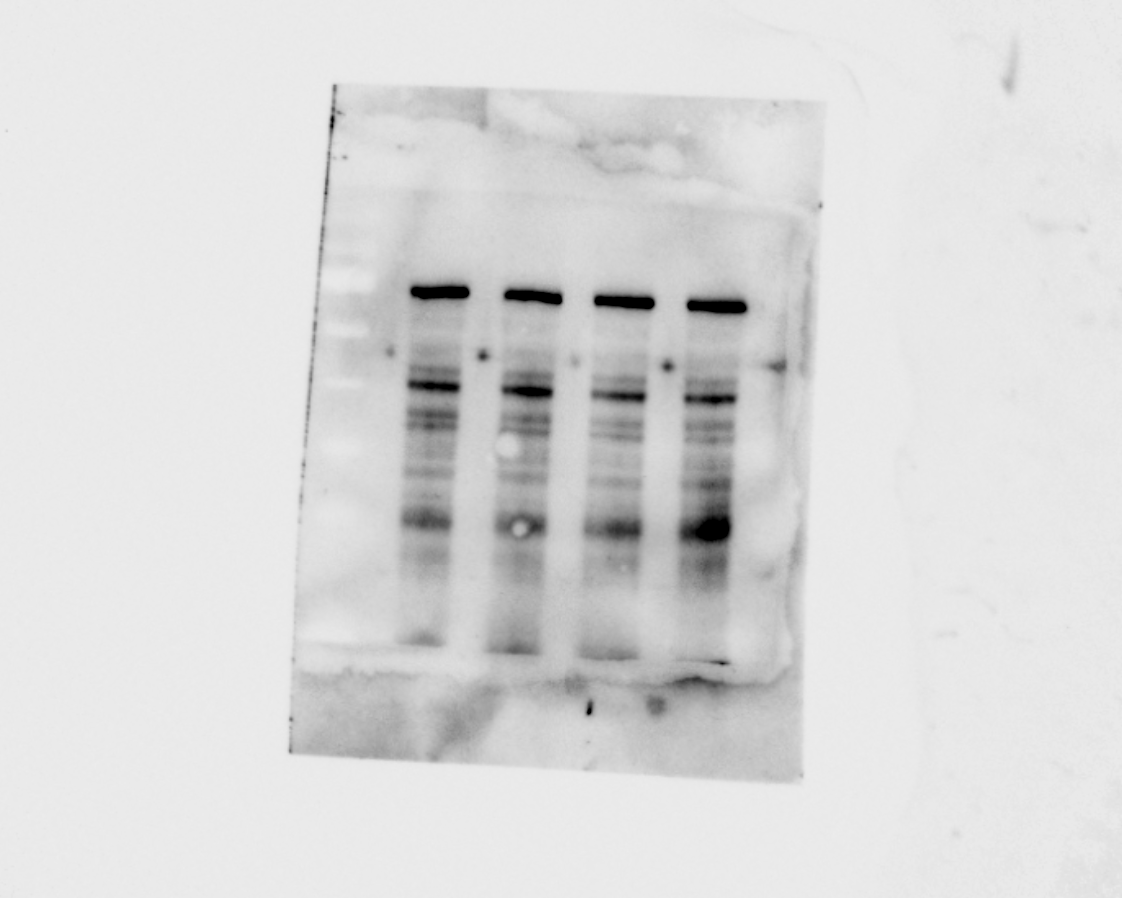

Supplement: Data S1 [file peerj-11-15643-s001.zip › ╨┬╜¿╬─╝■╝╨/(Ctrl,a┬-elemene,propranolol,a┬-elemene+propranolol) Akt,Erk,GAPDH(third).tif]

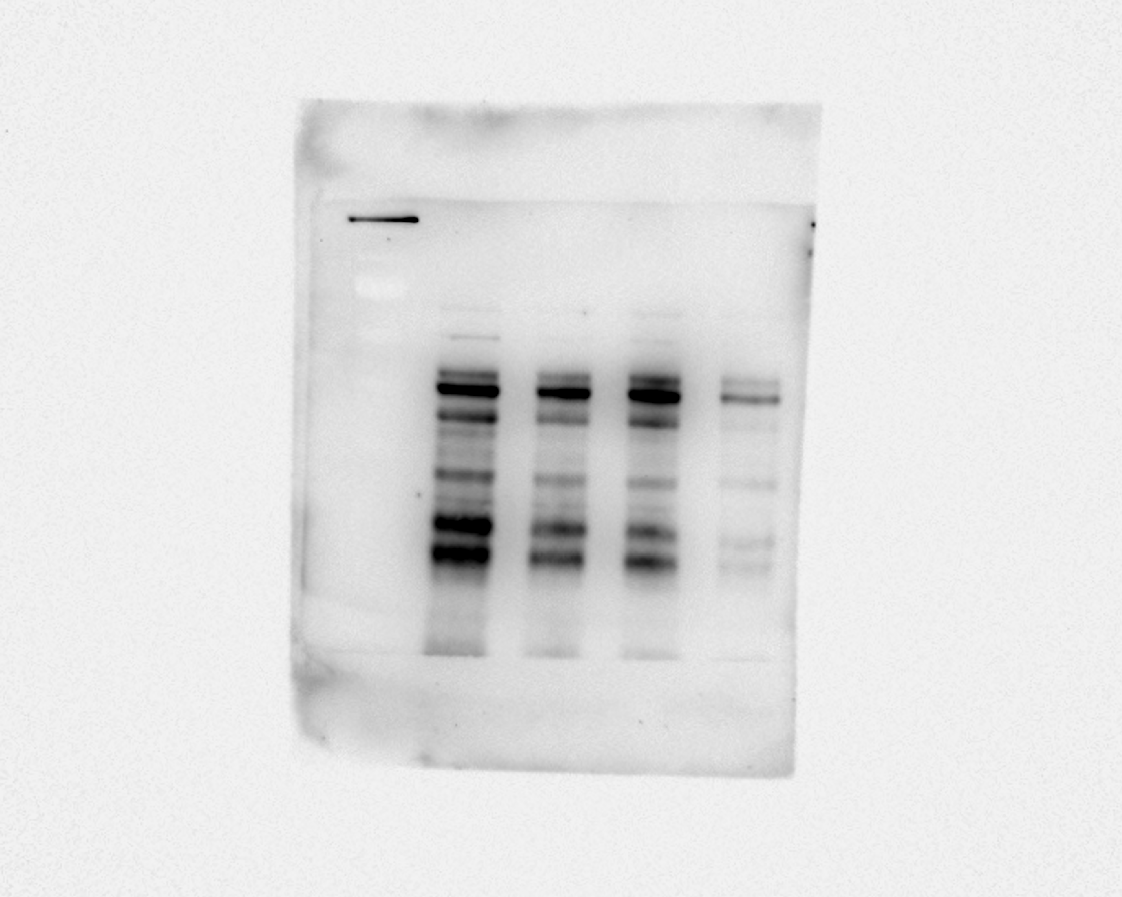

Supplement: Data S1 [file peerj-11-15643-s001.zip › ╨┬╜¿╬─╝■╝╨/(Ctrl,a┬-elemene,propranolol,a┬-elemene+propranolol) HIF-1-a┴, p-Akt,p-Erk,VEGFA(second).tif]

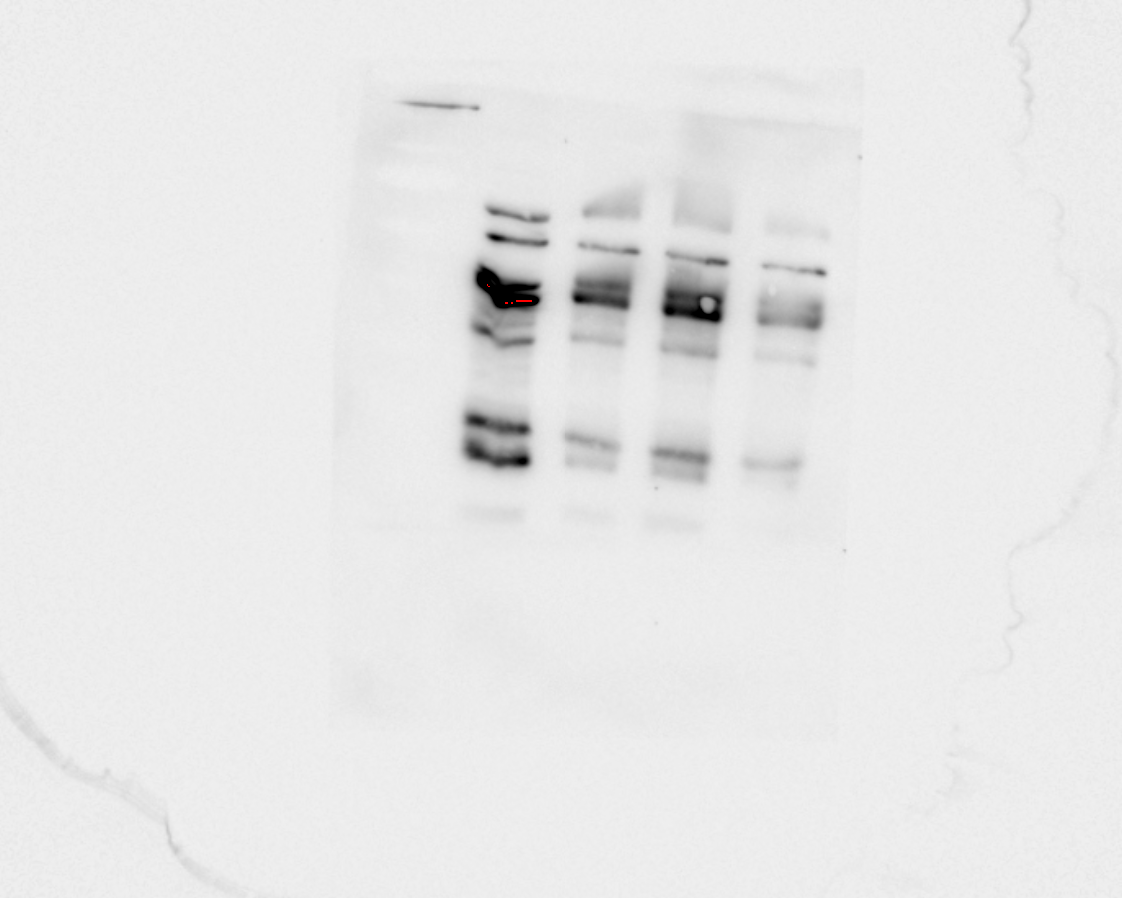

Supplement: Data S1 [file peerj-11-15643-s001.zip › ╨┬╜¿╬─╝■╝╨/(Ctrl,a┬-elemene,propranolol,a┬-elemene+propranolol) HIF-1-a┴, p-Akt,p-Erk,VEGFA(third).tif]

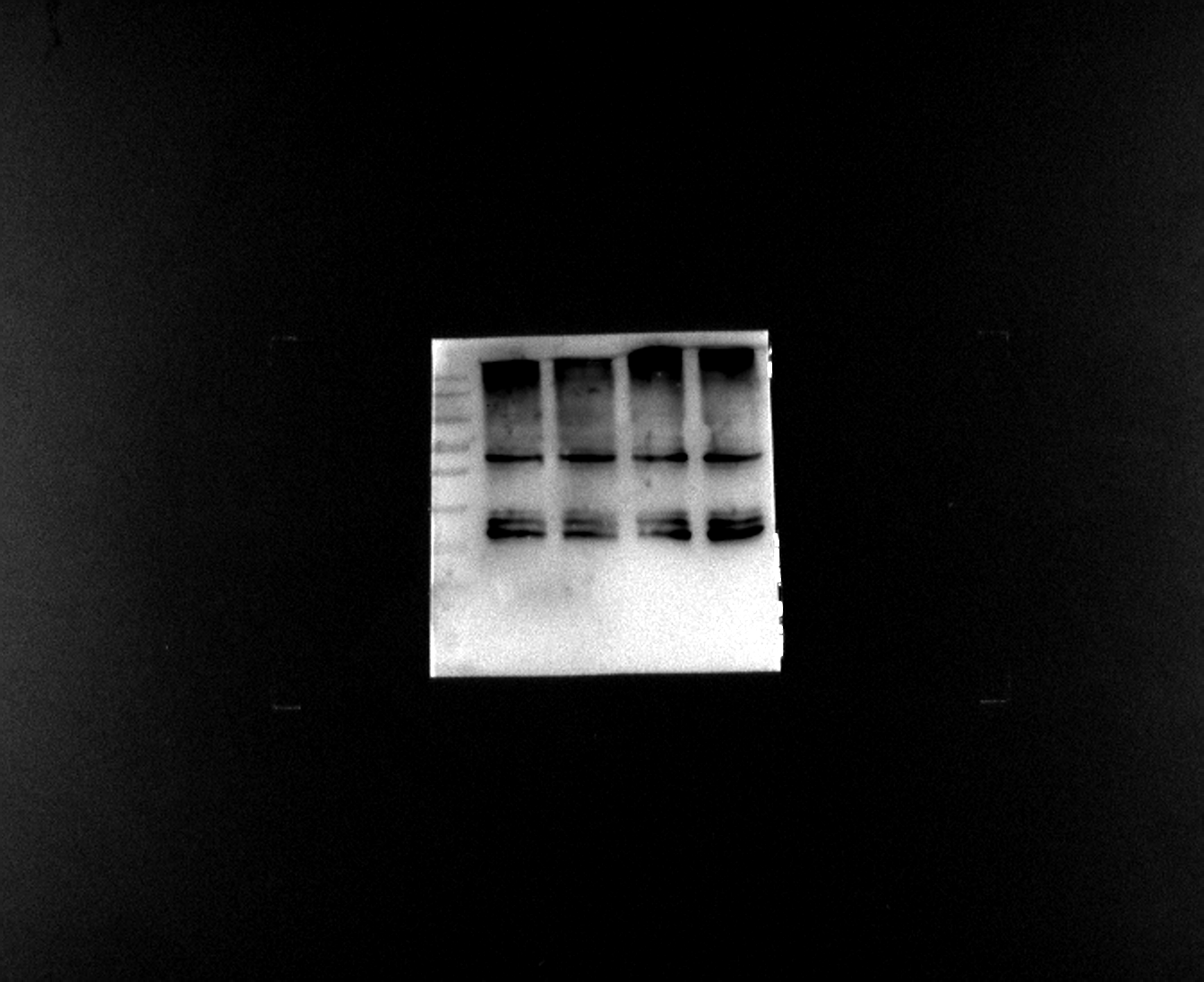

Supplement: Data S1 [file peerj-11-15643-s001.zip › ╨┬╜¿╬─╝■╝╨/(Ctrl,a┬-elemene,propranolol,a┬-elemene+propranolol)Akt,Erk.Tif]

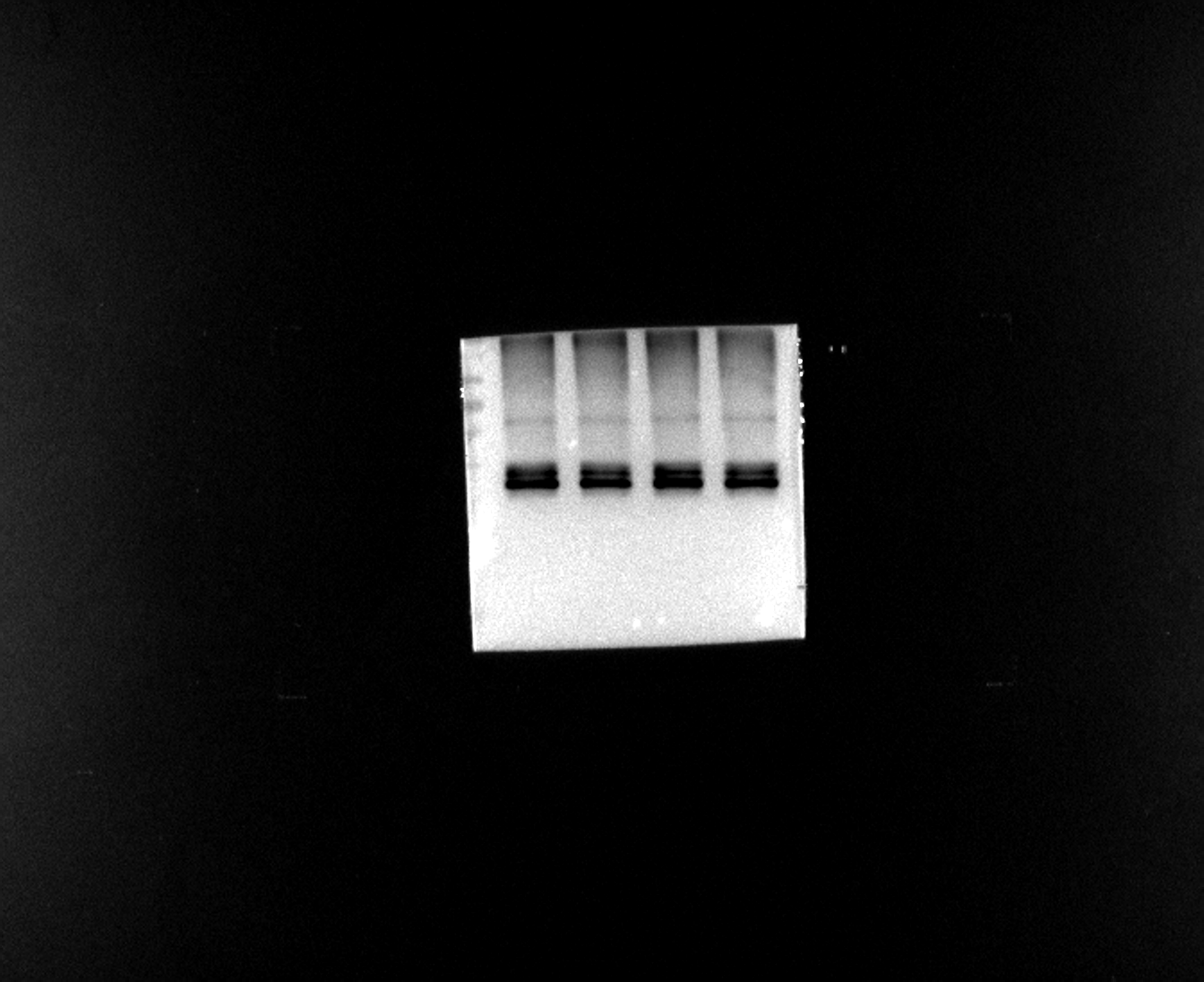

Supplement: Data S1 [file peerj-11-15643-s001.zip › ╨┬╜¿╬─╝■╝╨/(Ctrl,a┬-elemene,propranolol,a┬-elemene+propranolol)Erk.Tif]

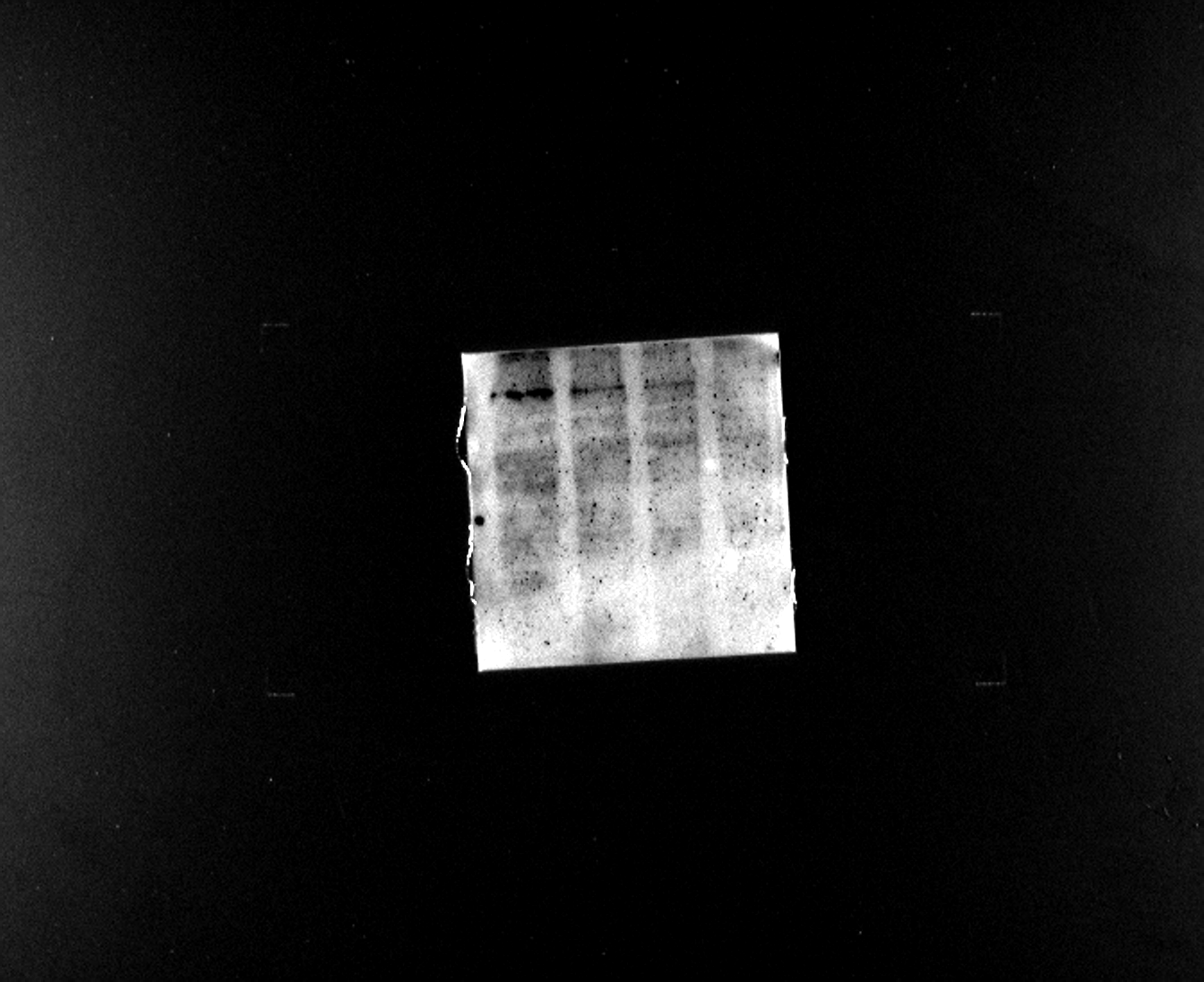

Supplement: Data S1 [file peerj-11-15643-s001.zip › ╨┬╜¿╬─╝■╝╨/(Ctrl,a┬-elemene,propranolol,a┬-elemene+propranolol)HIF-1-a┴.Tif]

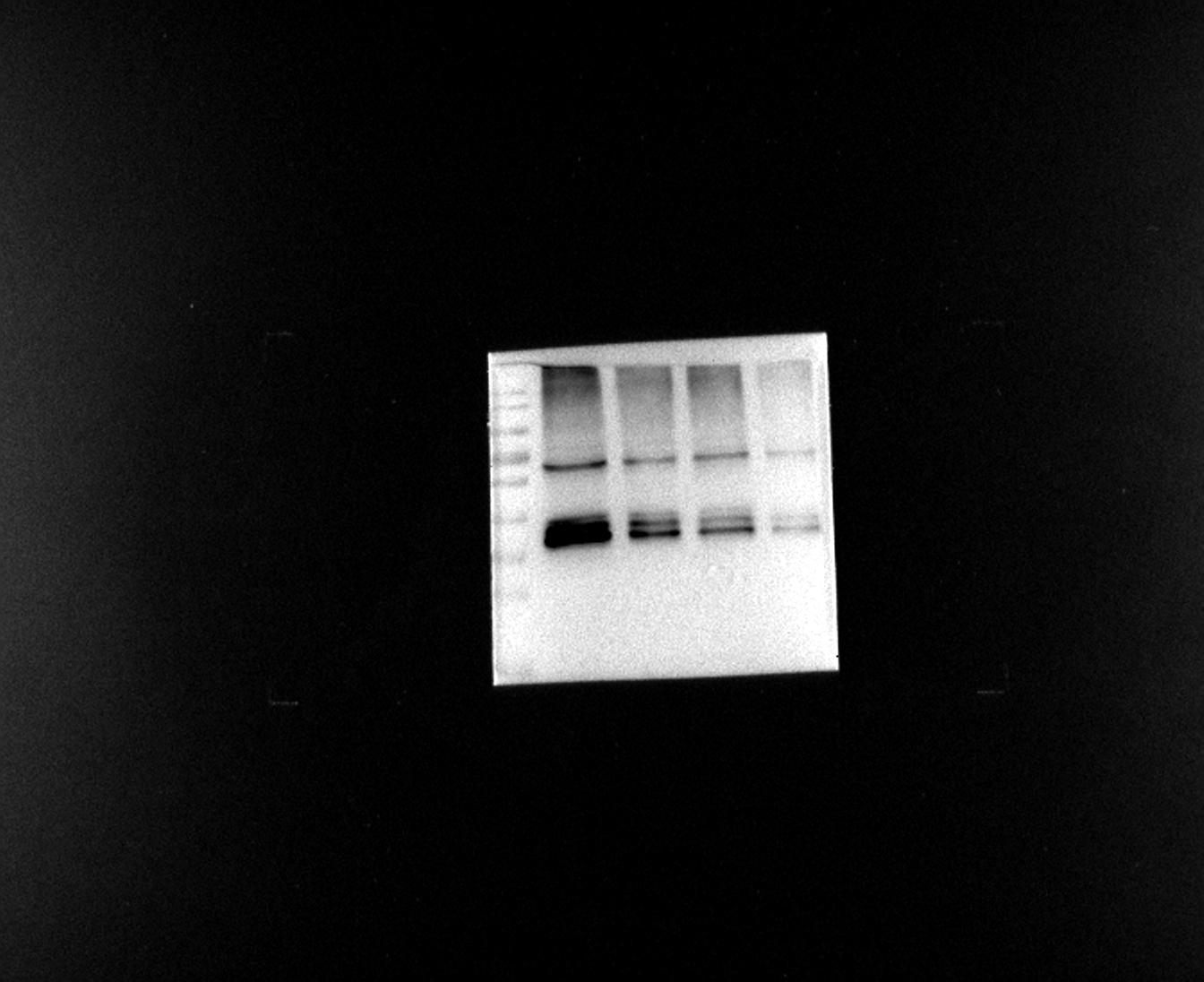

Supplement: Data S1 [file peerj-11-15643-s001.zip › ╨┬╜¿╬─╝■╝╨/(Ctrl,a┬-elemene,propranolol,a┬-elemene+propranolol)P-Akt,p-Erk.Tif]

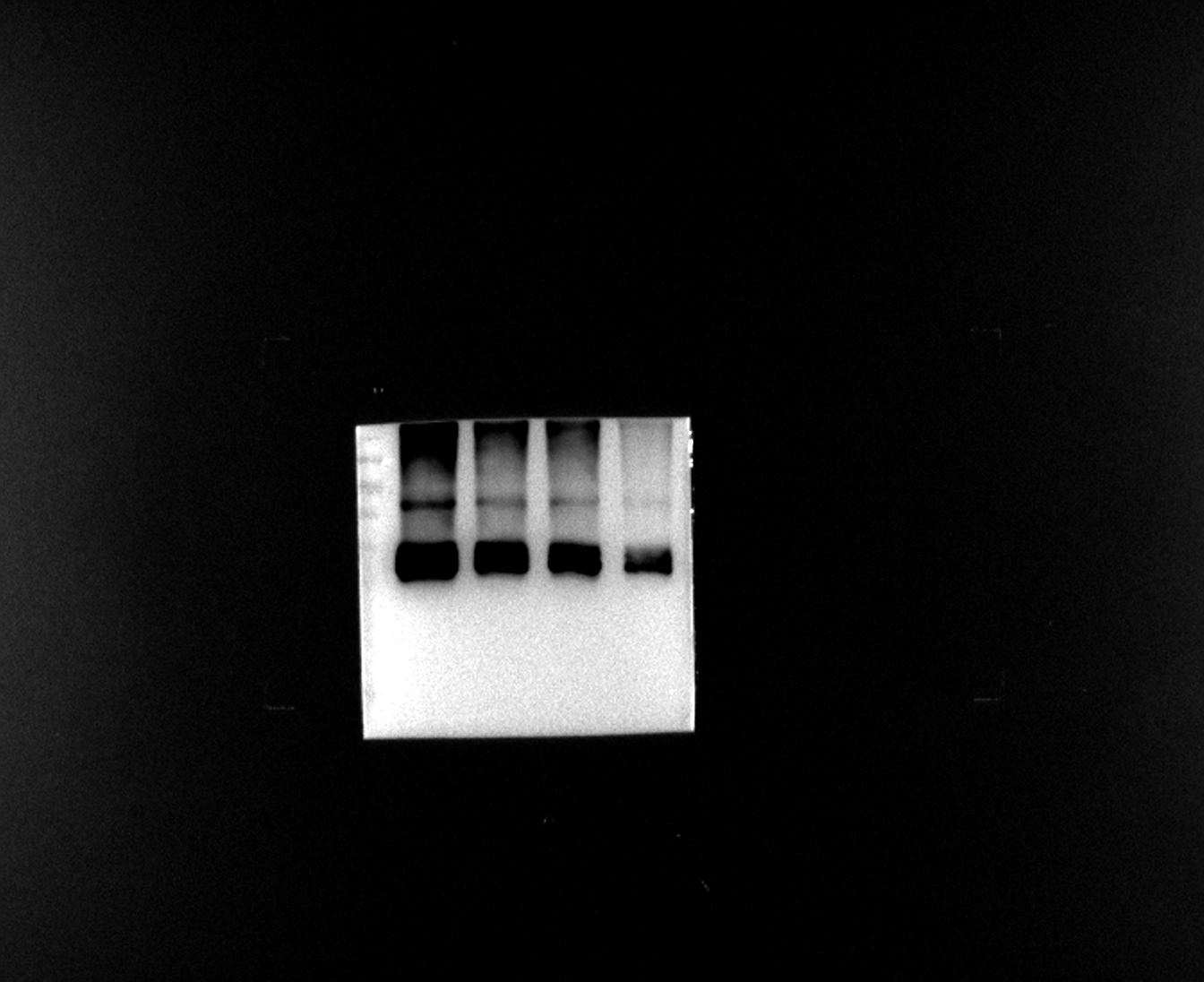

Supplement: Data S1 [file peerj-11-15643-s001.zip › ╨┬╜¿╬─╝■╝╨/(Ctrl,a┬-elemene,propranolol,a┬-elemene+propranolol)p-Akt.Tif]

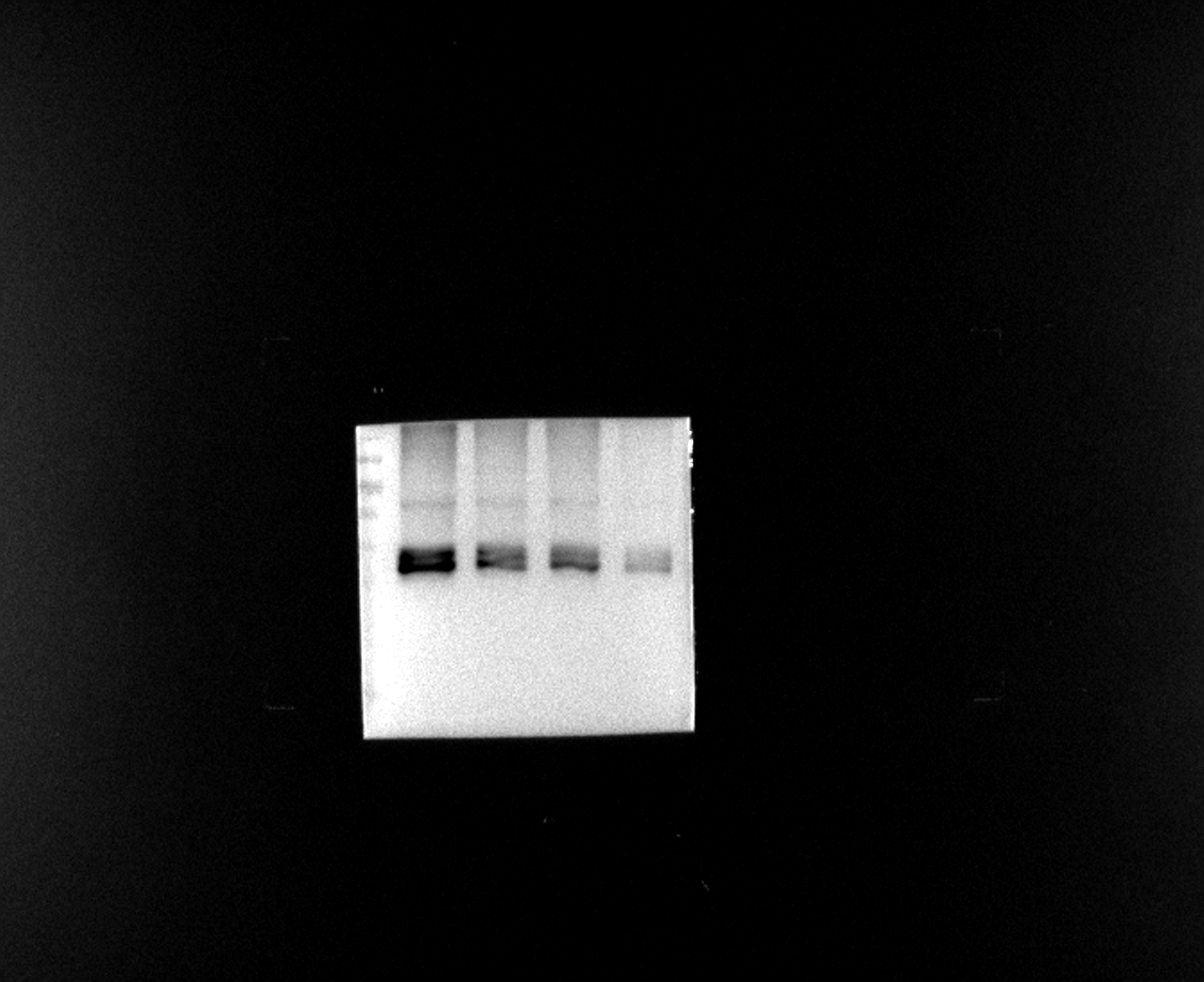

Supplement: Data S1 [file peerj-11-15643-s001.zip › ╨┬╜¿╬─╝■╝╨/(Ctrl,a┬-elemene,propranolol,a┬-elemene+propranolol)p-Erk.Tif]

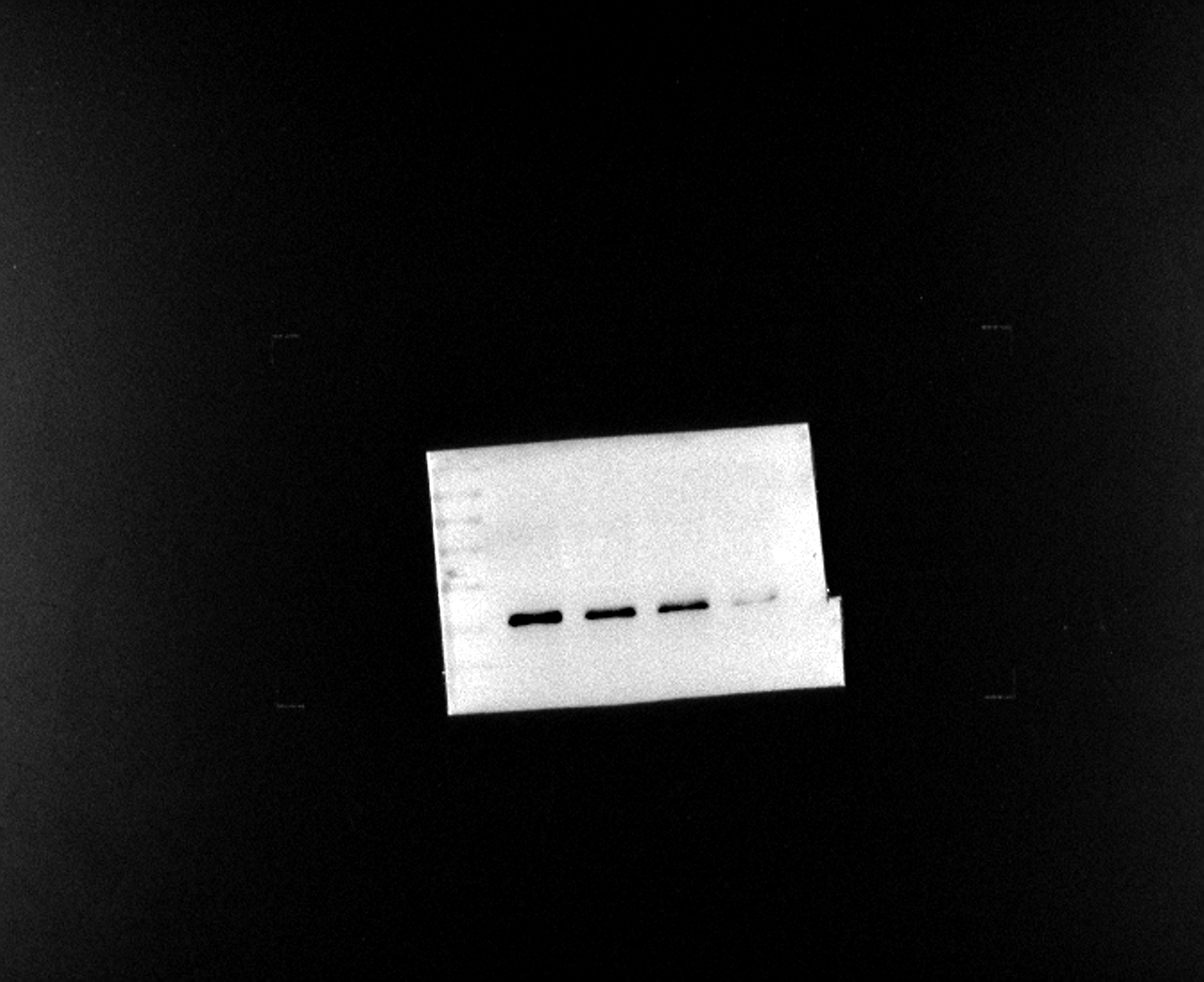

Supplement: Data S1 [file peerj-11-15643-s001.zip › ╨┬╜¿╬─╝■╝╨/(Ctrl,a┬-elemene,propranolol,a┬-elemene+propranolol)VEGFA.Tif]

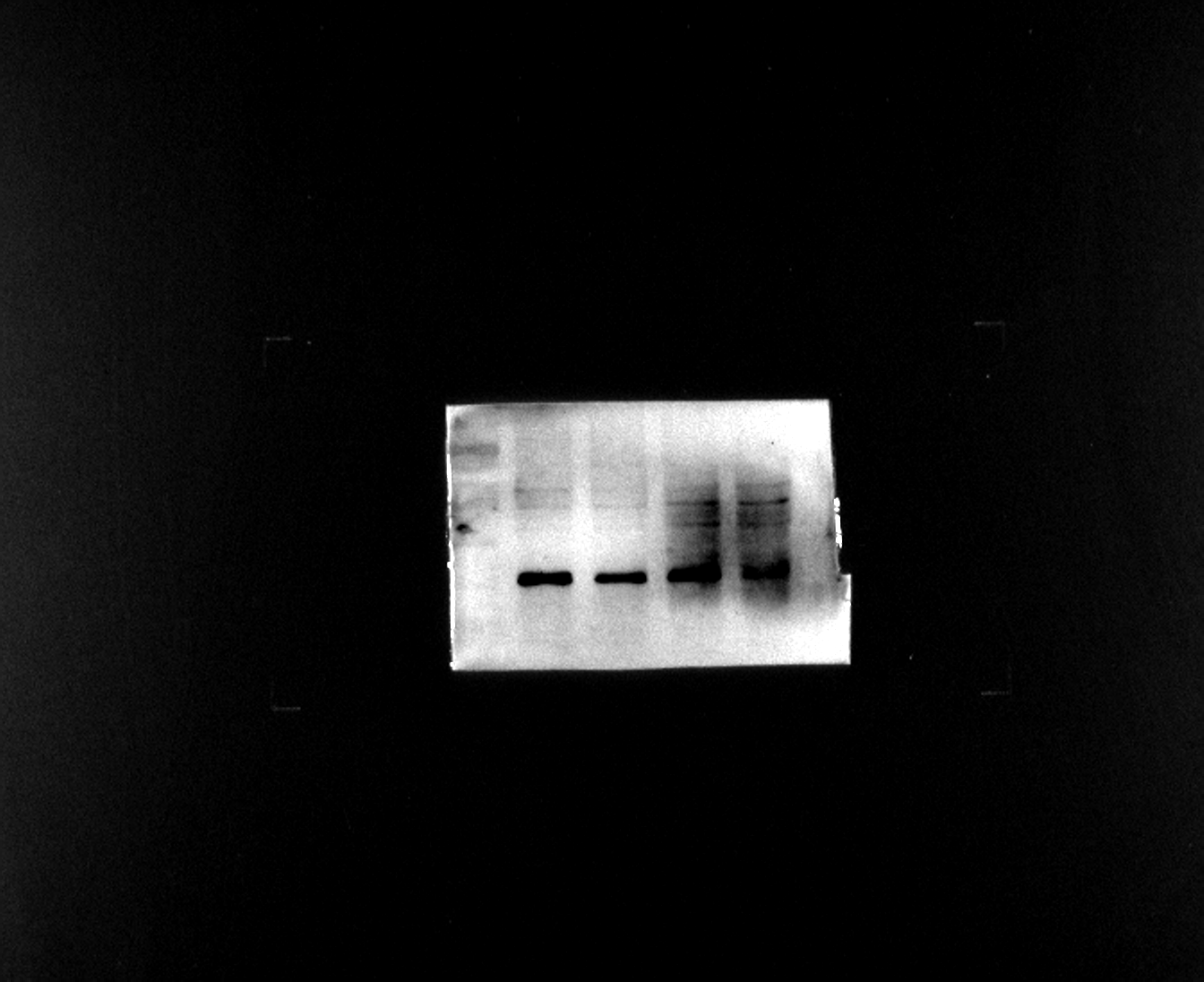

Supplement: Data S1 [file peerj-11-15643-s001.zip › ╨┬╜¿╬─╝■╝╨/(Ctrl,a┬-elemene,propranolol,a┬-elemene+propranolol)a┬-actin.Tif]

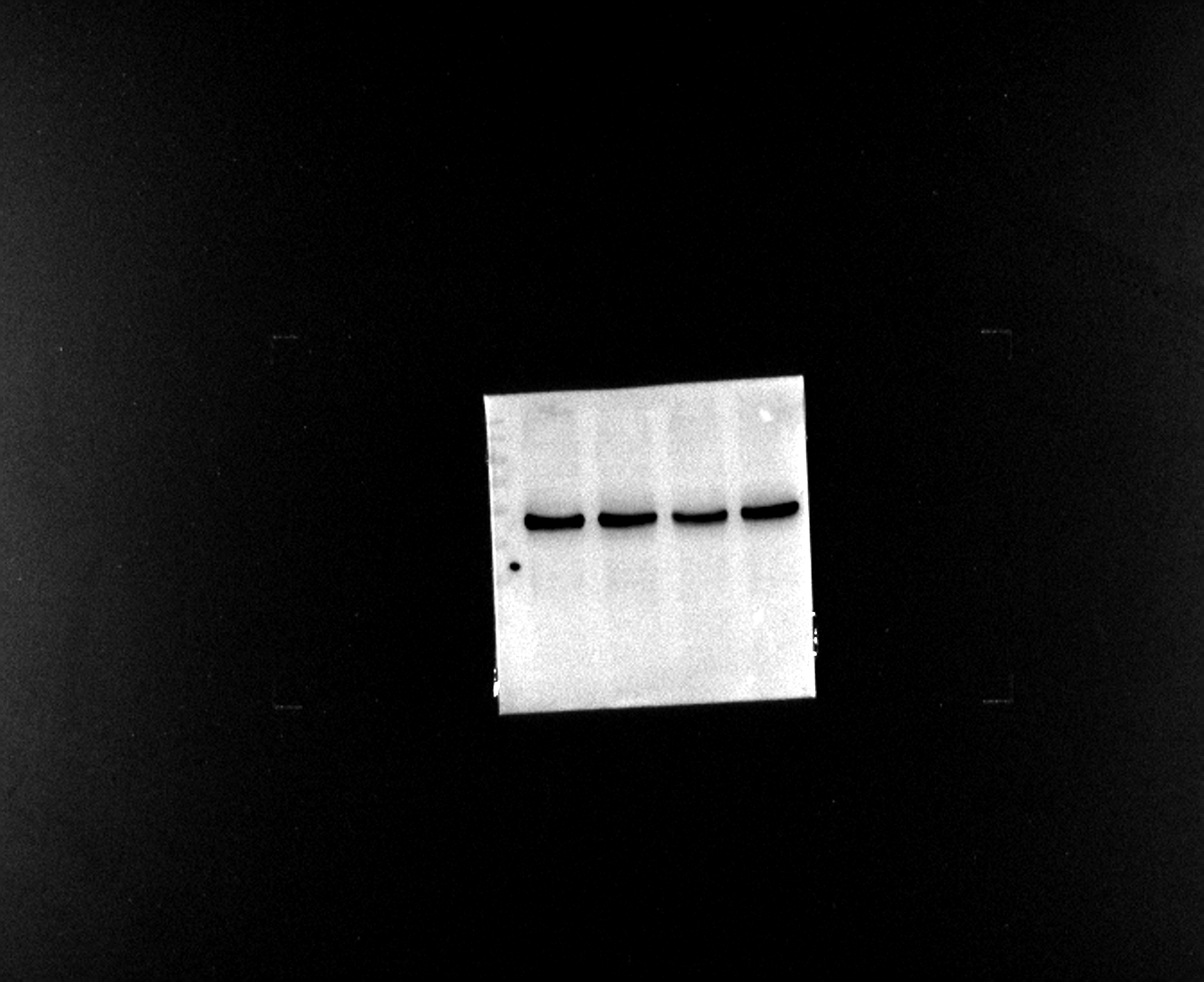

Supplement: Data S1 [file peerj-11-15643-s001.zip › ╨┬╜¿╬─╝■╝╨/(Ctrl,a┬-elemene,propranolol,a┬-elemene+propranolol)a┬-Tubulin.Tif]

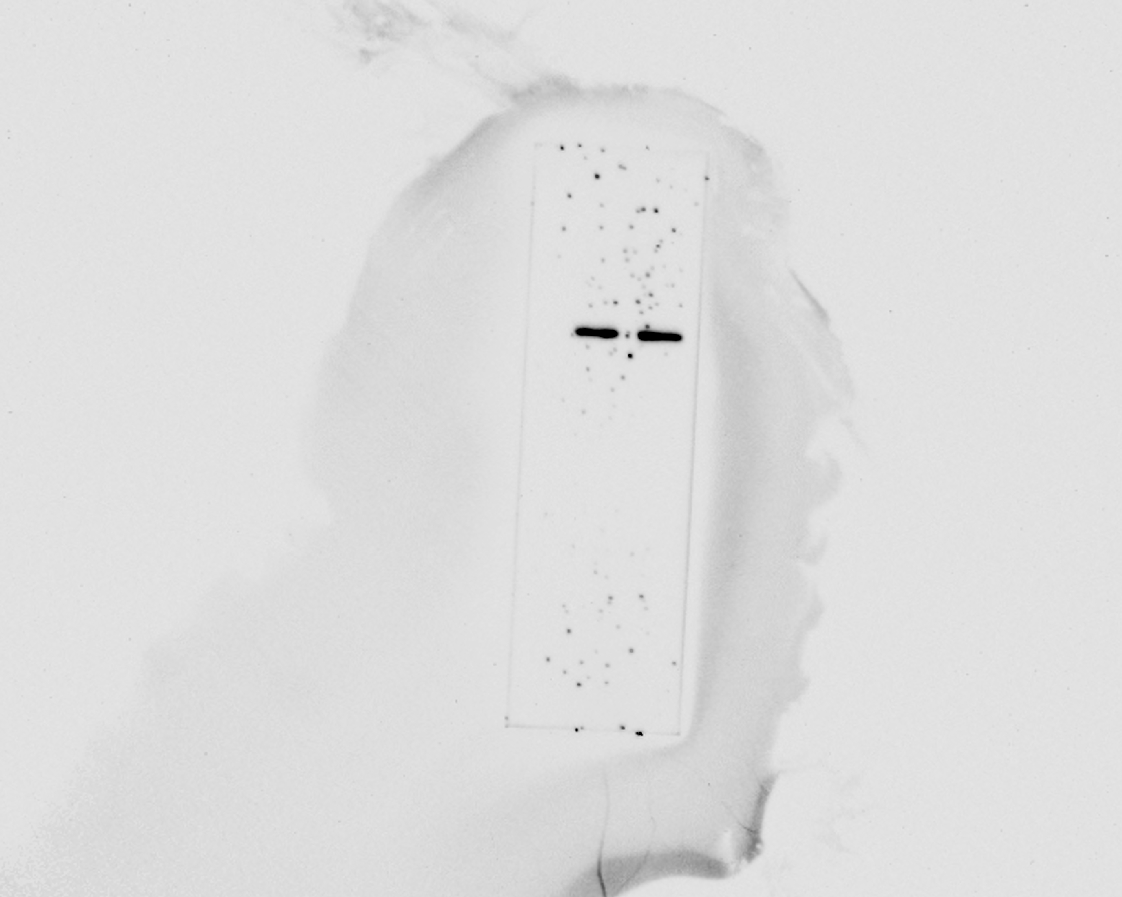

Supplement: Data S1 [file peerj-11-15643-s001.zip › ╨┬╜¿╬─╝■╝╨/(HUVECs vs HemECs)Akt(first).jpg]

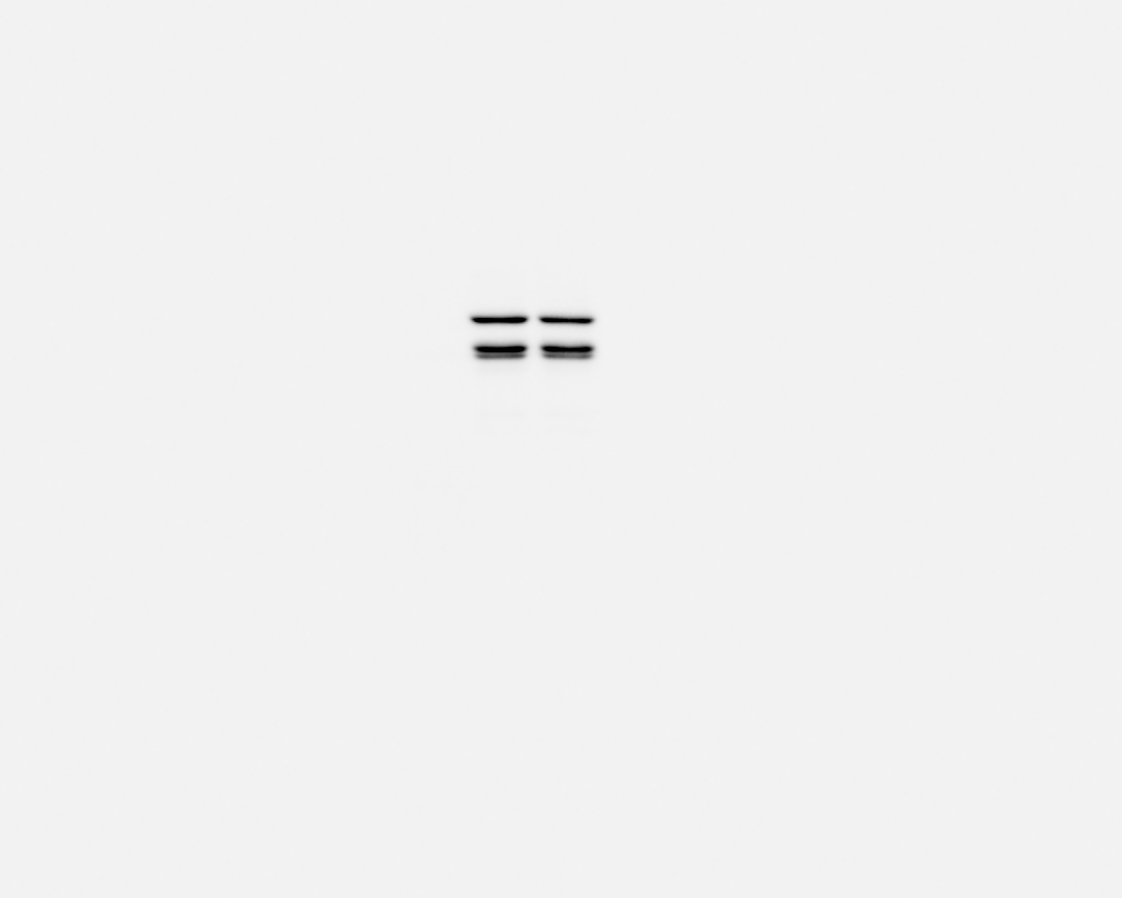

Supplement: Data S1 [file peerj-11-15643-s001.zip › ╨┬╜¿╬─╝■╝╨/(HUVECs vs HemECs)Akt,Erk(second).tif]

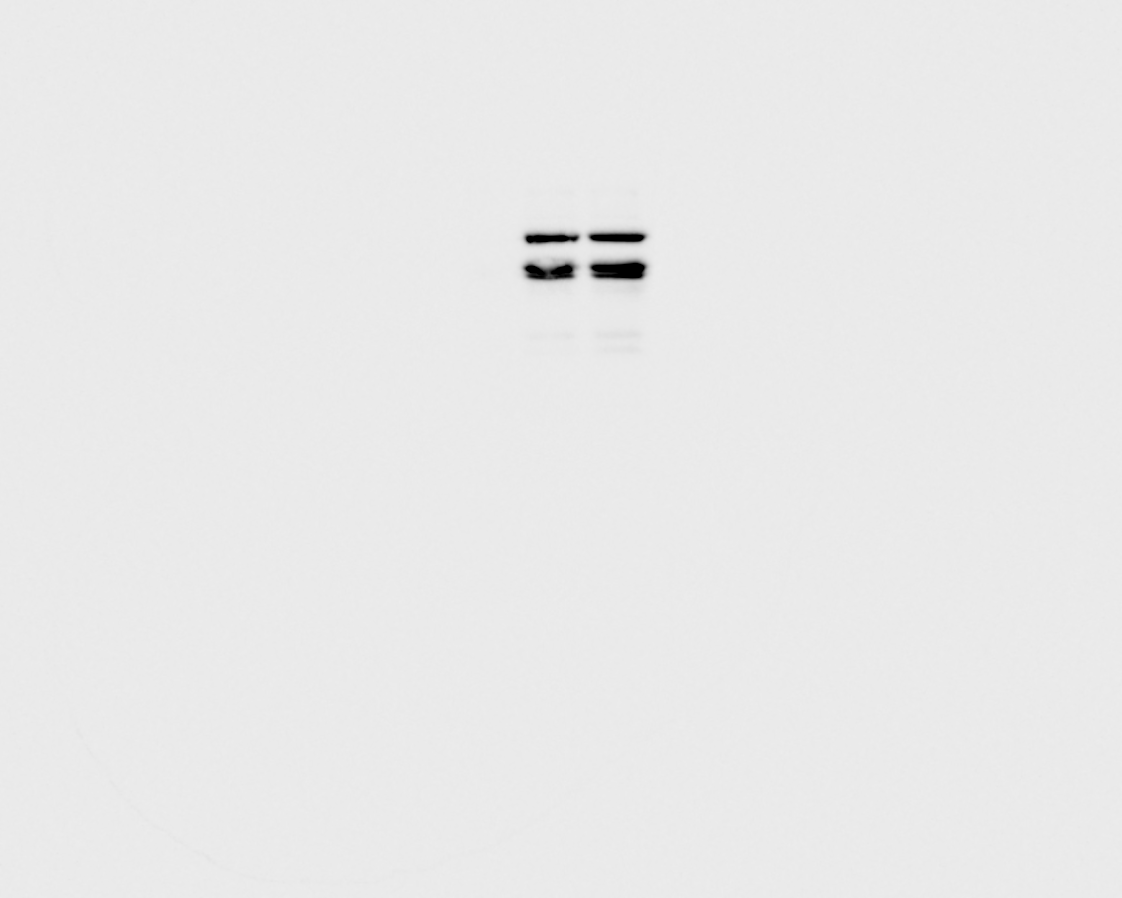

Supplement: Data S1 [file peerj-11-15643-s001.zip › ╨┬╜¿╬─╝■╝╨/(HUVECs vs HemECs)Akt,Erk(third).tif]

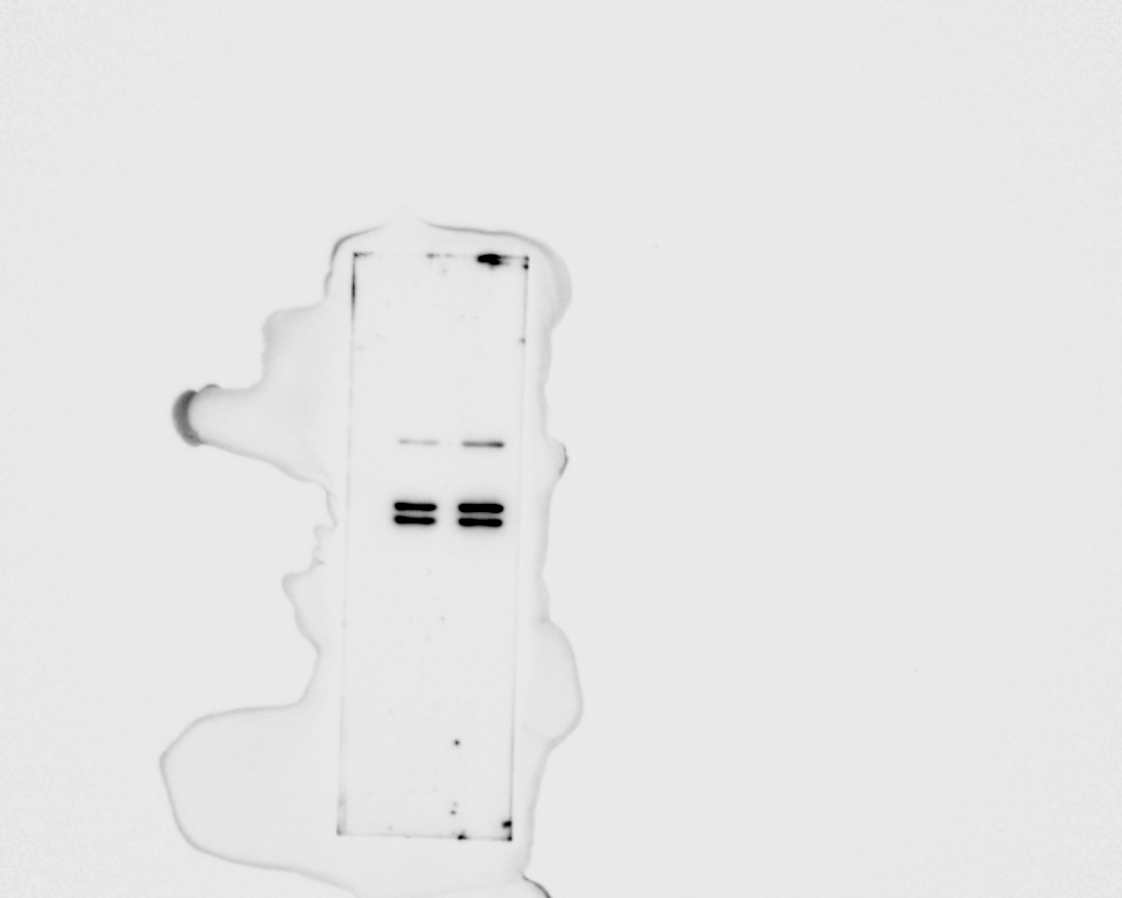

Supplement: Data S1 [file peerj-11-15643-s001.zip › ╨┬╜¿╬─╝■╝╨/(HUVECs vs HemECs)Erk(first).tif]

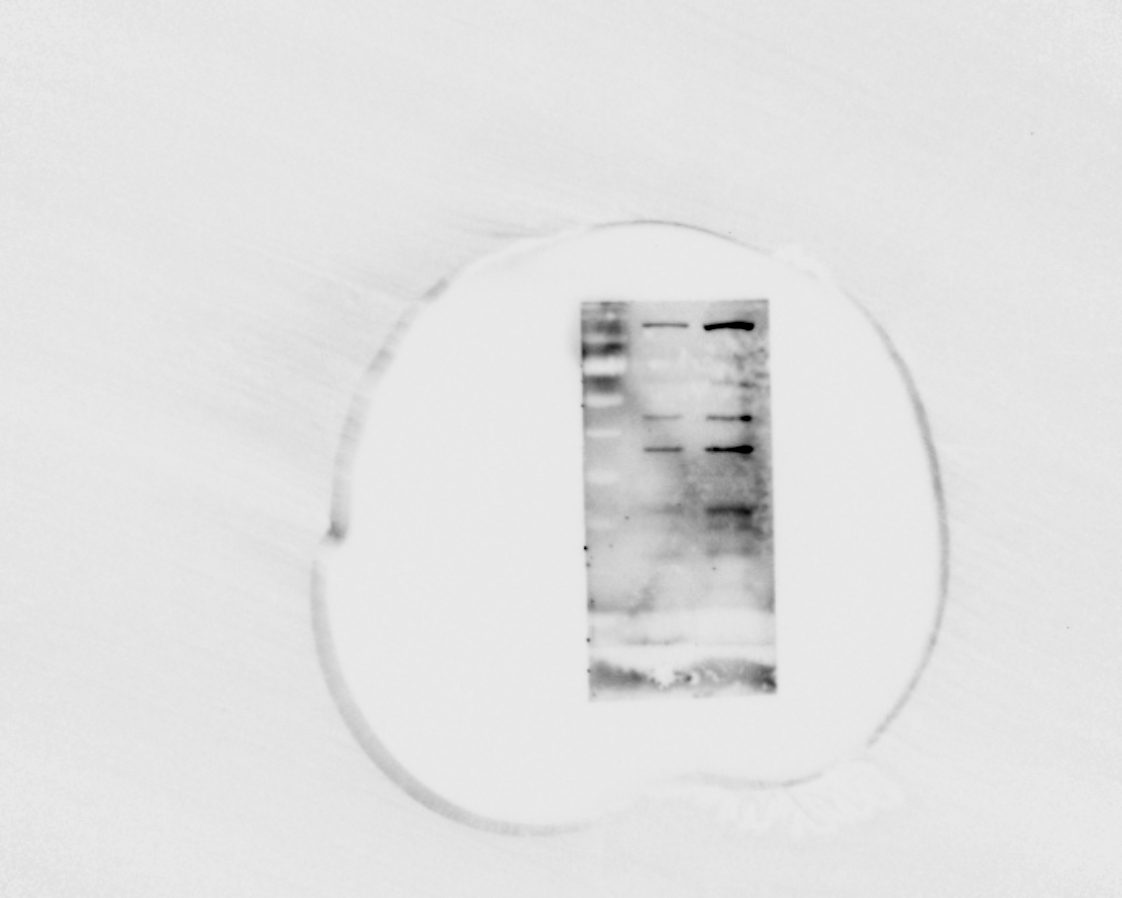

Supplement: Data S1 [file peerj-11-15643-s001.zip › ╨┬╜¿╬─╝■╝╨/(HUVECs vs HemECs)HIF-1-a┴(third).tif]

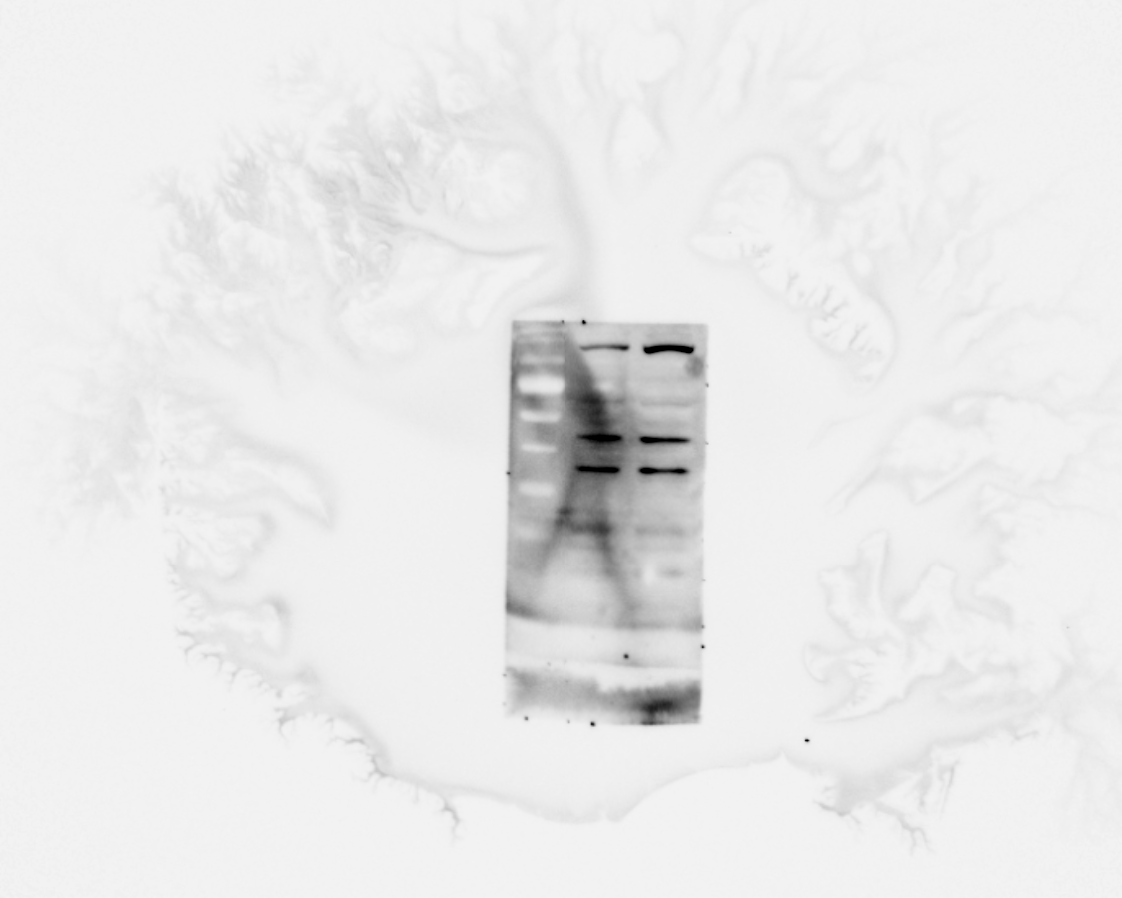

Supplement: Data S1 [file peerj-11-15643-s001.zip › ╨┬╜¿╬─╝■╝╨/(HUVECs vs HemECs)HIF-1-a┴,a┬-actin(second).tif]

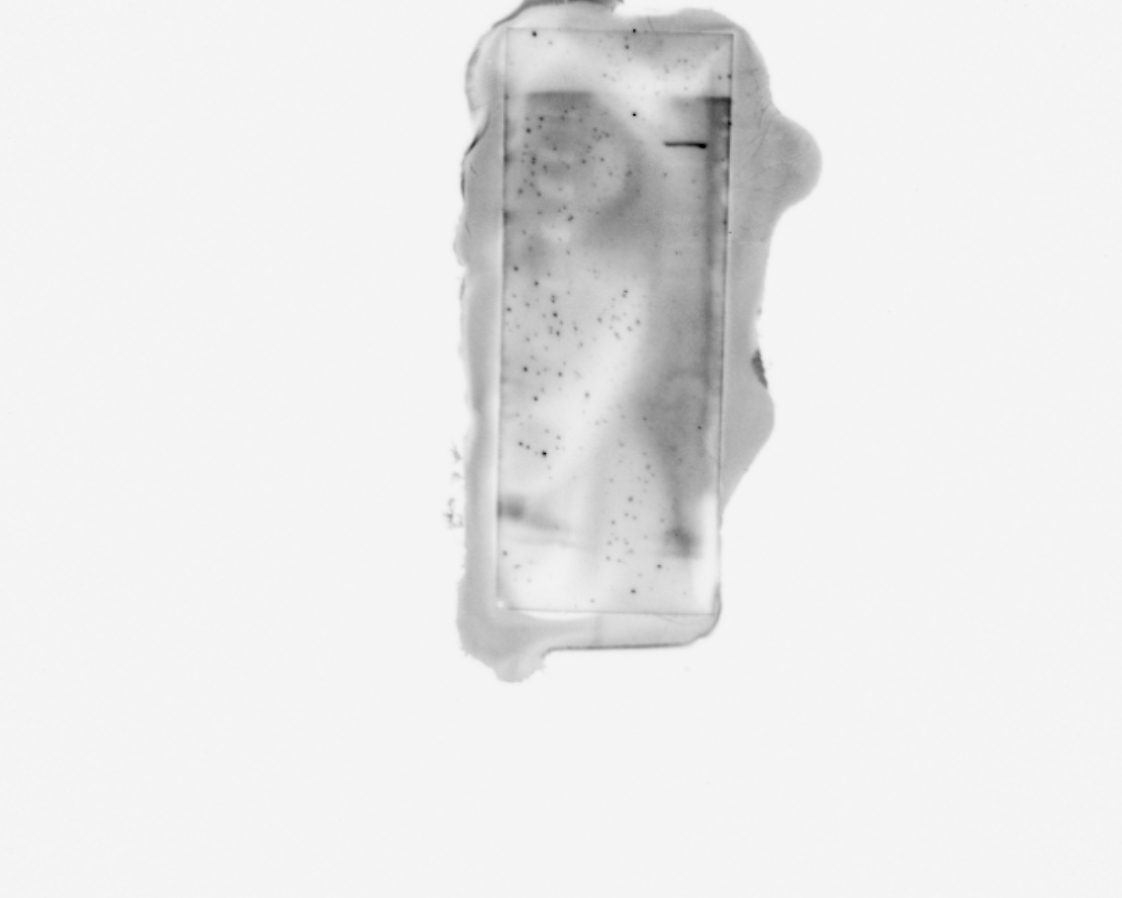

Supplement: Data S1 [file peerj-11-15643-s001.zip › ╨┬╜¿╬─╝■╝╨/(HUVECs vs HemECs)HIF-1-a┴.jpg]

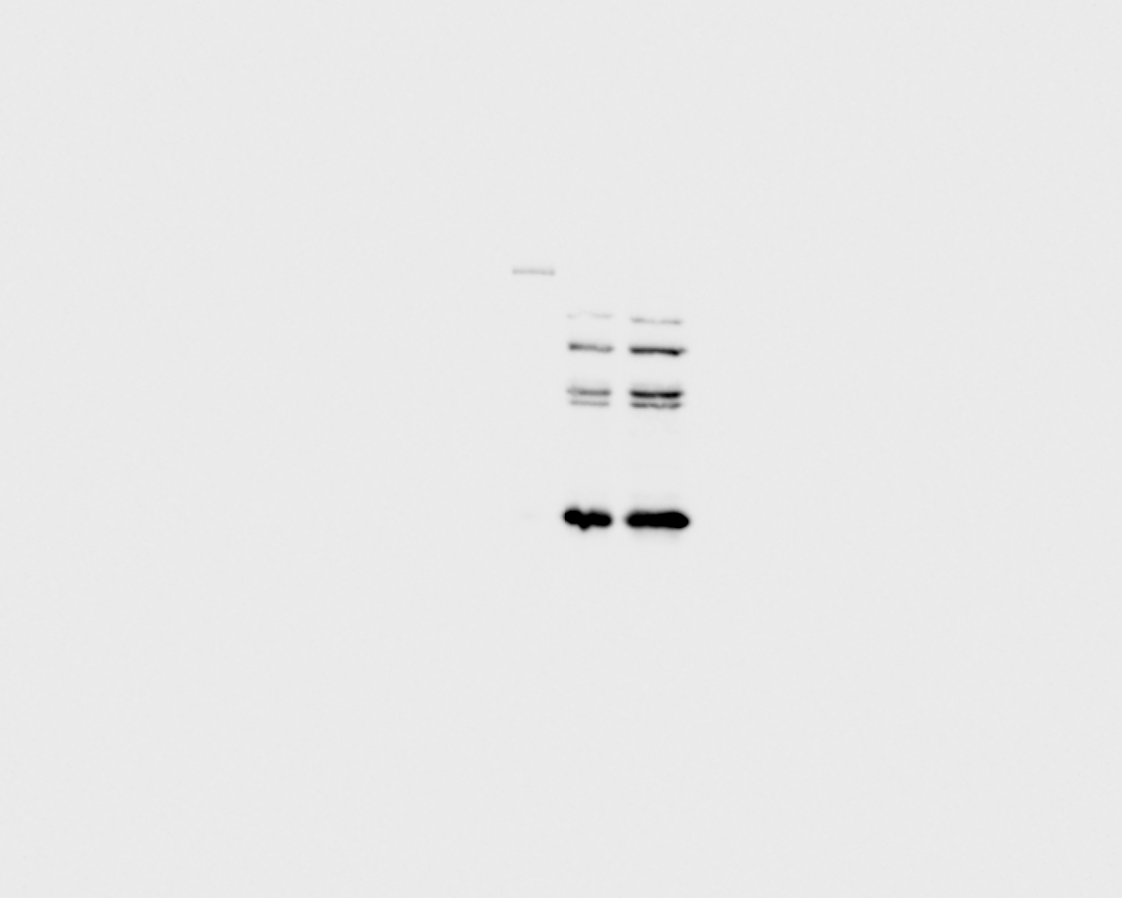

Supplement: Data S1 [file peerj-11-15643-s001.zip › ╨┬╜¿╬─╝■╝╨/(HUVECs vs HemECs)p-Akt,p-Erk,VEGFA(second).tif]

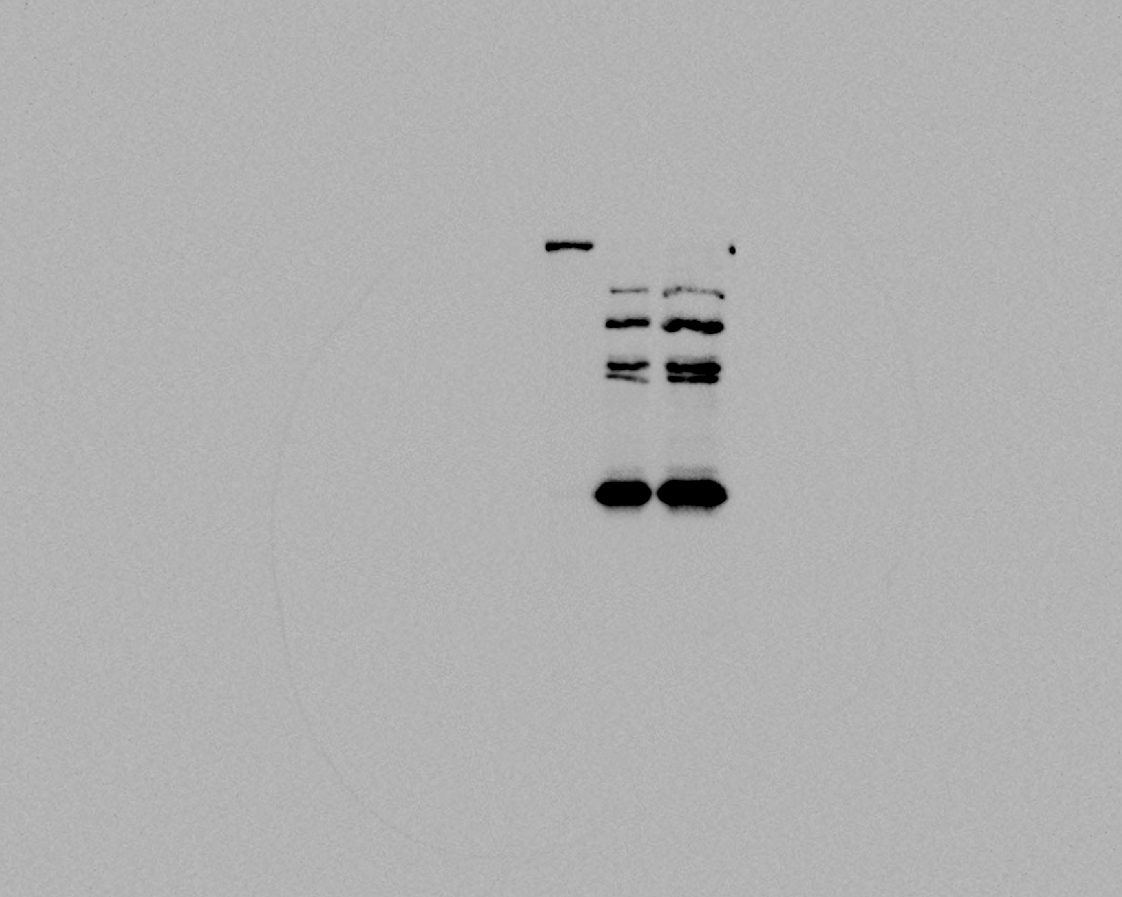

Supplement: Data S1 [file peerj-11-15643-s001.zip › ╨┬╜¿╬─╝■╝╨/(HUVECs vs HemECs)p-Akt,p-Erk,VEGFA(third).tif]

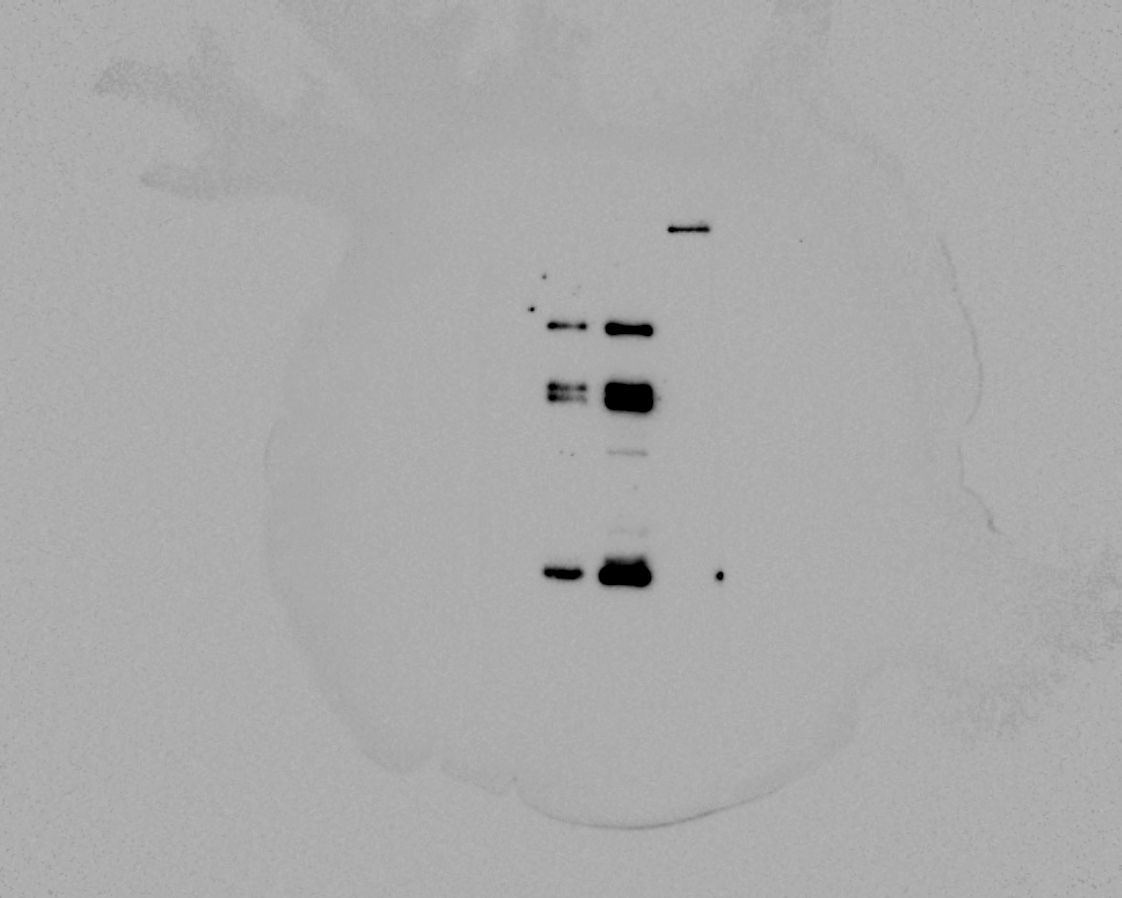

Supplement: Data S1 [file peerj-11-15643-s001.zip › ╨┬╜¿╬─╝■╝╨/(HUVECs vs HemECs)p-Akt,p-Erk,VEGFA.tif]

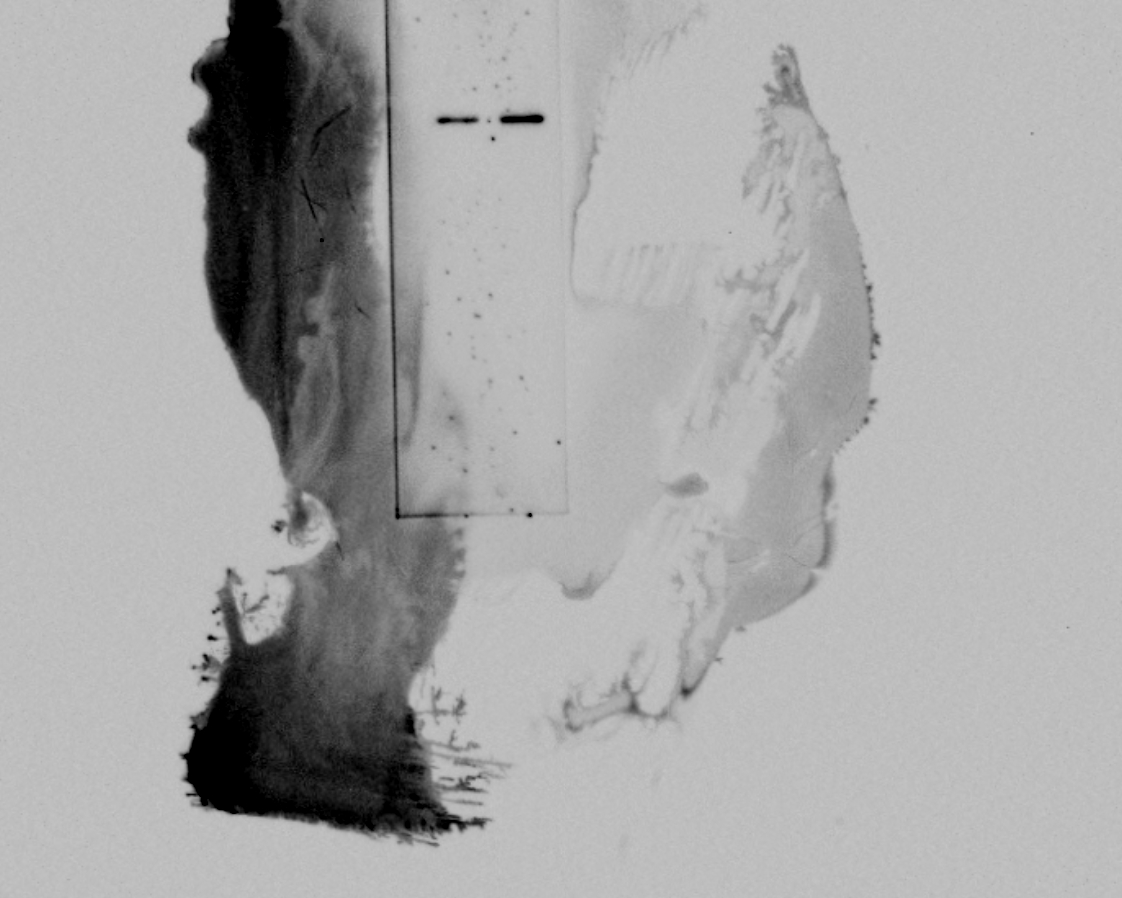

Supplement: Data S1 [file peerj-11-15643-s001.zip › ╨┬╜¿╬─╝■╝╨/(HUVECs vs HemECs)p-Akt.jpg]

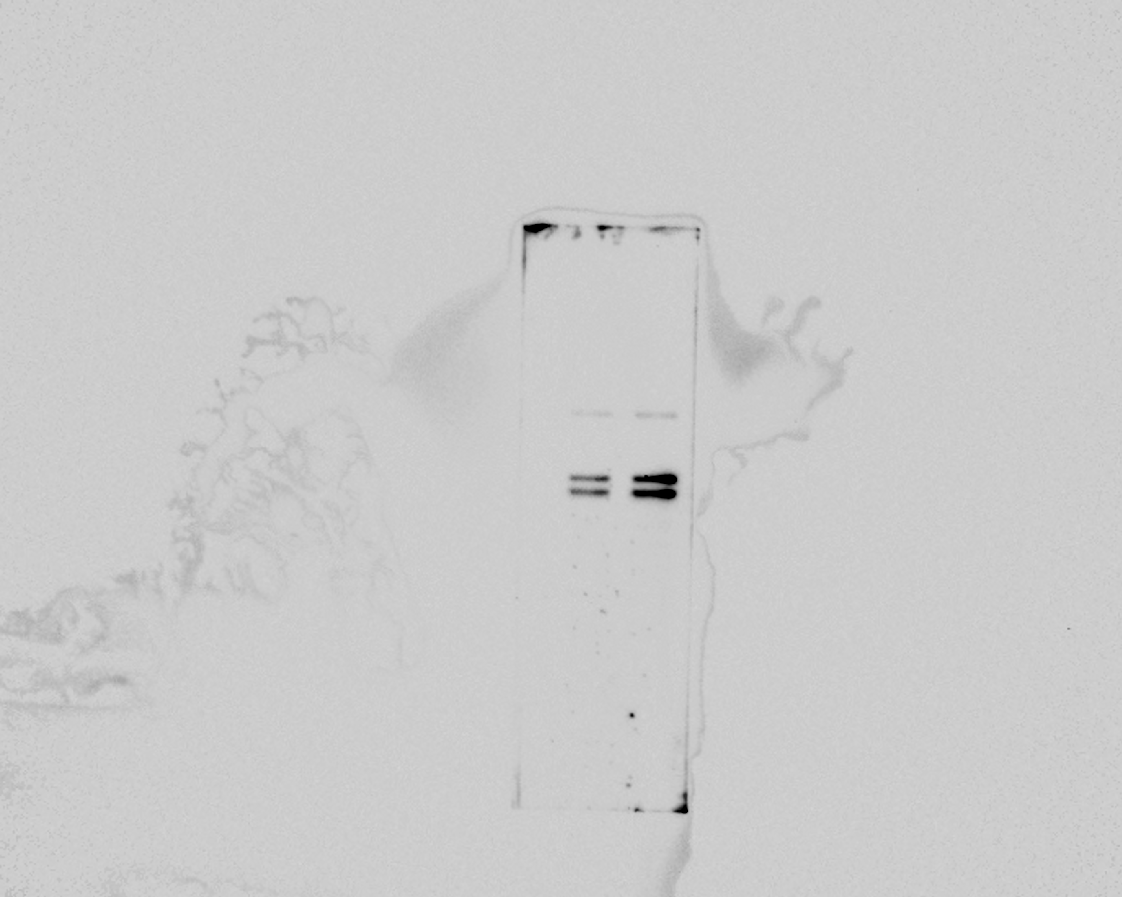

Supplement: Data S1 [file peerj-11-15643-s001.zip › ╨┬╜¿╬─╝■╝╨/(HUVECs vs HemECs)p-Erk.tif]

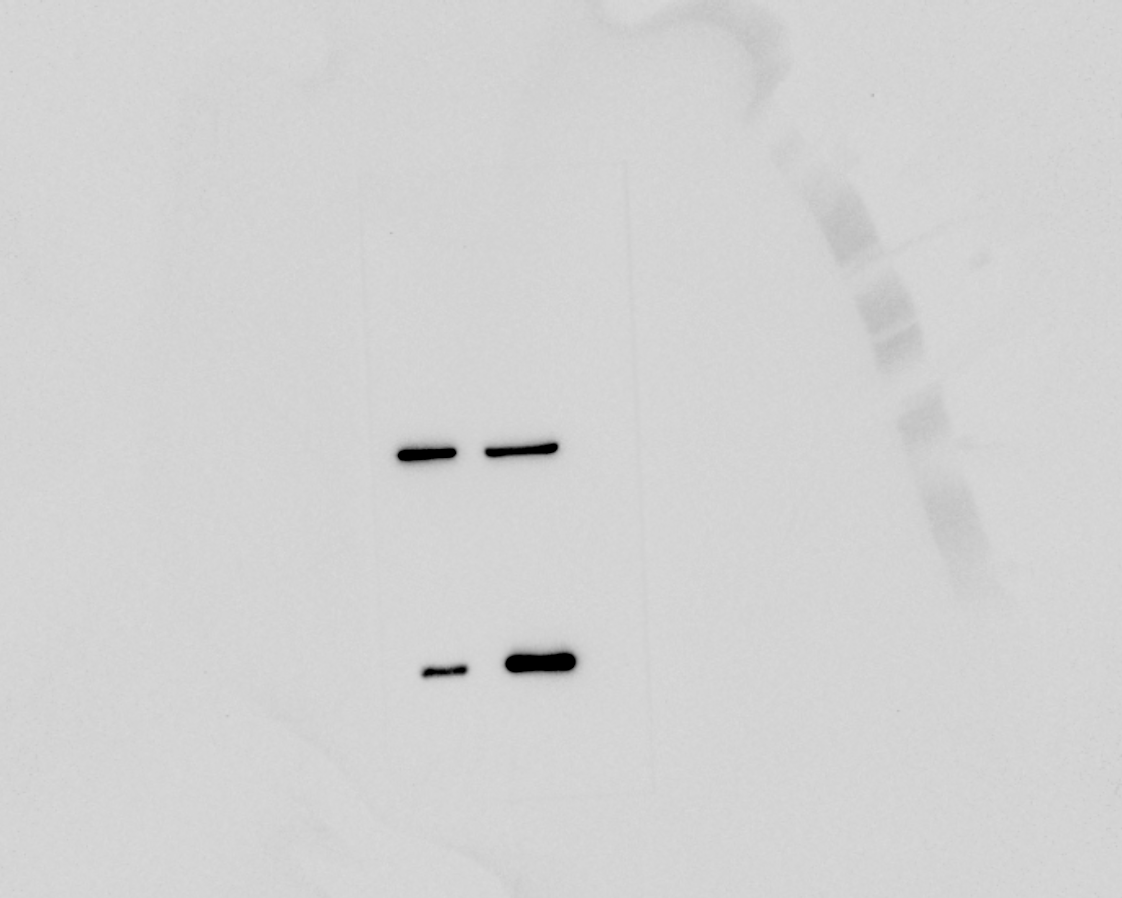

Supplement: Data S1 [file peerj-11-15643-s001.zip › ╨┬╜¿╬─╝■╝╨/(HUVECs vs HemECs)VEGFA,a┬Tubulin.tif]

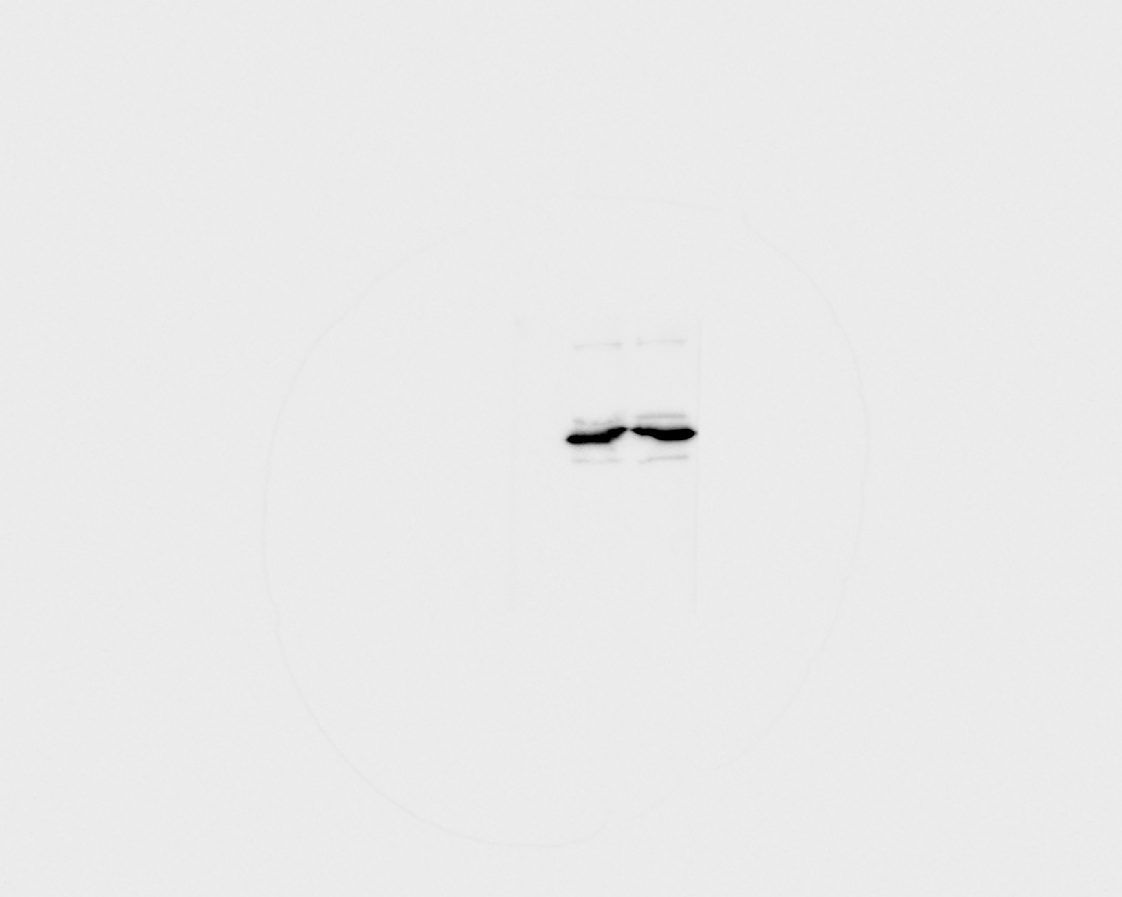

Supplement: Data S1 [file peerj-11-15643-s001.zip › ╨┬╜¿╬─╝■╝╨/(HUVECs vs HemECs)a┬Tubulin(second).tif]

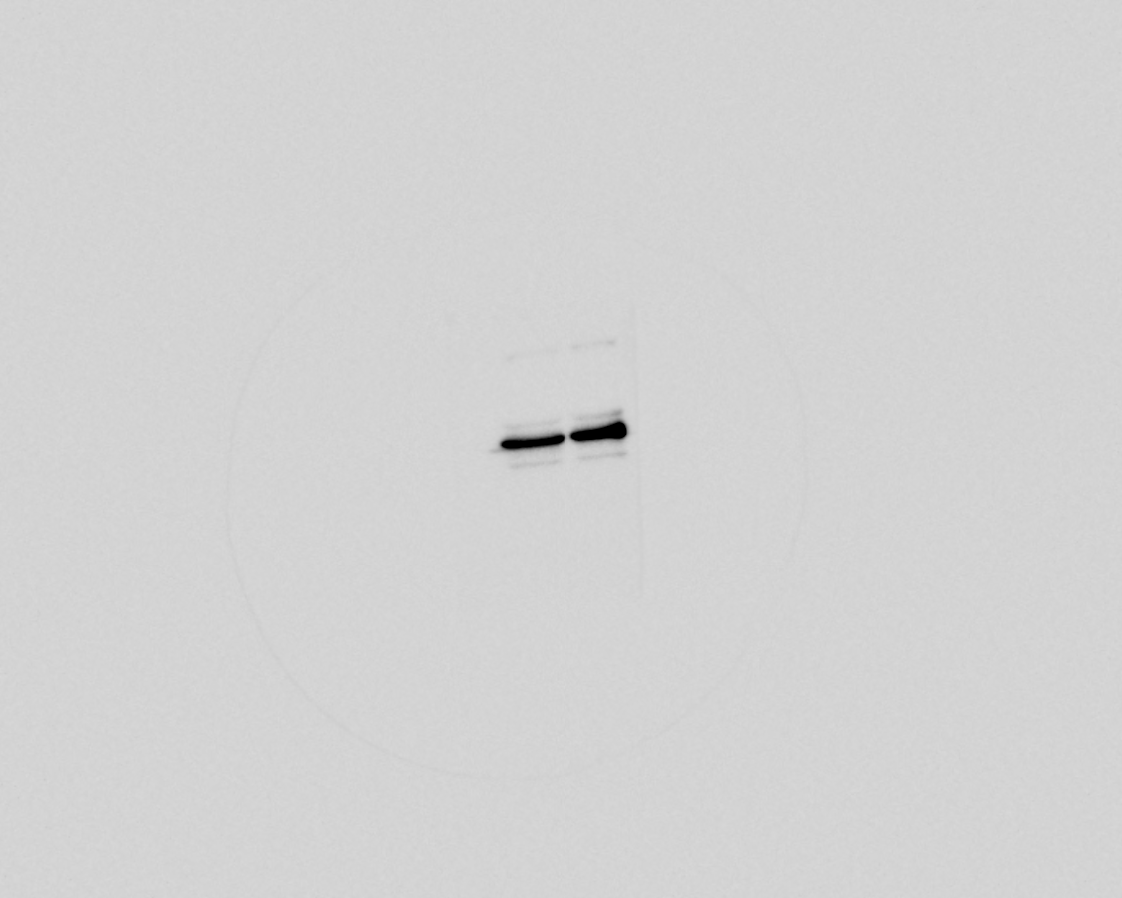

Supplement: Data S1 [file peerj-11-15643-s001.zip › ╨┬╜¿╬─╝■╝╨/(HUVECs vs HemECs)a┬Tubulin(third).tif]

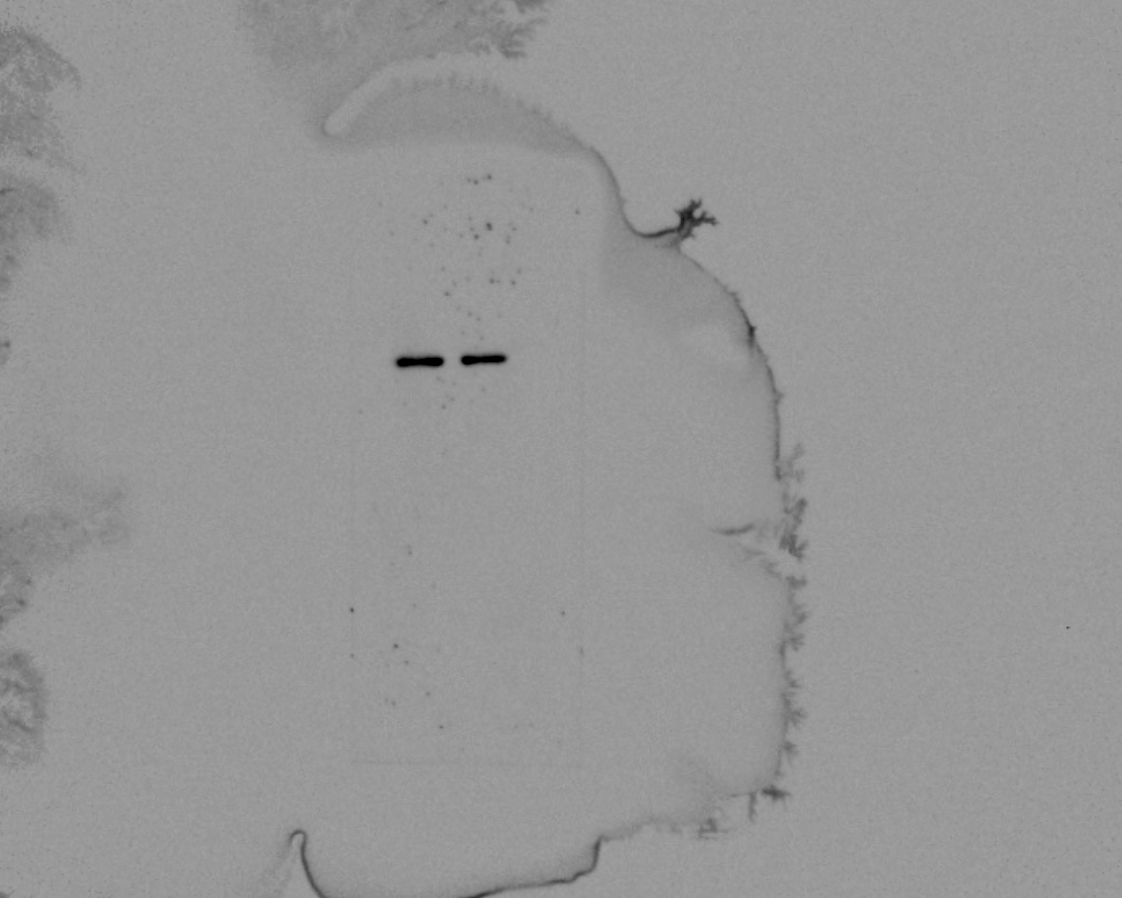

Supplement: Data S1 [file peerj-11-15643-s001.zip › ╨┬╜¿╬─╝■╝╨/(HUVECs vs HemECs)a┬Tubulin.tif]
